# Forum theatre as a tool to promote positive donkey welfare on Lamu Island, Kenya

**DOI:** 10.1017/awf.2025.12

**Published:** 2025-03-14

**Authors:** Emily Haddy, Leanne Proops, Tamsin Bradley, Cressida Bowyer, Obadiah Sing’Oei

**Affiliations:** 1 Faculty of Humanities and Social Sciences, University of Portsmouth, Portsmouth, UK; 2 School of Psychology, Sport and Health Sciences, University of Portsmouth, Portsmouth, UK; 3 Faculty of Creative and Cultural Industries, University of Portsmouth, Portsmouth, UK; 4 The Donkey Sanctuary, Lamu Island, Kenya

**Keywords:** animal welfare, community engagement, donkey, drama, forum theatre, participatory, methods, working equid welfare

## Abstract

When targeting human behaviour change for animal welfare improvement, engaging with communities is vital. Equid-reliant communities are often resource poor, geographically isolated and disparities in literacy rates are common, presenting challenges to ‘traditional’ forms of engagement. Arts-based initiatives using non-written communication methods such as storytelling and performance, may be ideal media to convey positive welfare messages. In this study we evaluate the feasibility of using forum theatre to sensitise donkey-reliant communities regarding key welfare issues. Through a co-creation process, a piece of interactive forum theatre on donkey welfare was produced and staged for the public and in local schools. Post-performance questionnaire data were collected from adults and both pre- and post-performance data in schools to evaluate changes in knowledge and attitudes resulting from the performance. Quantitative and qualitative data were collected using Likert scales and open questions, respectively. Audience feedback was positive, with more than 90% of audiences strongly agreeing that they enjoyed the performance. More than 85% of adult respondents strongly agreed that the performance raised their awareness of three key indicators: donkey health needs; donkey welfare needs; and how much donkeys should carry. For youth audiences, comparison of pre- and post-performance measures demonstrated positive changes in the belief that donkeys feel pain, how much individuals liked donkeys and how confident they felt in identifying how a donkey was feeling. Although participatory arts-based approaches remain rare in the animal welfare sector, the study highlights the potential value of these methods in promoting community engagement for positive animal welfare changes.

## Introduction

Globally, it is thought that 112 million working equids support the lives and livelihoods of more than 600 million people in low and middle income countries (LMICs), although it is acknowledged that these figures are likely to be largely underestimated (Norris *et al.* 2021). Working equids provide an essential role in generating income for families across a broad range of industries as well as providing access to basic necessities (Pritchard *et al.* 2005; Pritchard 2010). They also offer a social lifeline, providing economic resilience, security and income diversification to many vulnerable groups, including marginalised communities, women and those in extreme poverty described as the ‘bottom billion’ of the world’s population (Stringer 2014; Geiger *et al.* 2020; Watson *et al.* 2020). However, despite their vital role, levels of working equid welfare are generally poor, with global data highlighting a high prevalence of welfare problems and a lack of representation in animal health and agricultural policy (Burn *et al.* 2010; Pritchard 2010). As a consequence of compromised health, equid working performance and productivity is negatively impacted, limiting the support and income generation they can provide (Tesfaye & Curran 2005).

In response to global need, equid welfare non-governmental organisations (NGOs) utilise a variety of programmatic approaches to improve working equid welfare and support the people that rely upon them. Approaches can encompass the provision of free or subsidised veterinary care, providing education and training in equine-related skills, enhancing local infrastructure and advocacy campaigning (Upjohn *et al.* 2014; Haddy *et al.* 2022). Regardless of the type of initiative in place, improvements in animal welfare will rely upon changing human behaviour and it is acknowledged that engaging with communities is vital to the success of welfare programmes (Pritchard *et al.* 2018; Reed & Upjohn 2018). However, every community is unique and this engagement needs to be tailored to meet the specific needs of the target community. This involves ensuring that any information presented is in a format that is suitable both culturally and across levels of understanding in the community in order to have impact (Stringer *et al.* 2011).

Working equid-reliant communities can present challenges to traditionally utilised forms of engagement; they are typically resource poor, geographically isolated and access to education is often limited resulting in disparities in literacy rates (Stringer *et al.* 2011). Arts-based initiatives, visual art, storytelling and especially performance are not dependent upon high literacy levels and are thought to have wide general appeal, breaking down traditional research hierarchies, and gaining access to a large number of sometimes difficult to reach stakeholders (Bunn *et al.* 2020). Arts-based initiatives may therefore be an ideal medium in which to engage with communities and convey positive welfare messages.

Theatre is one such art form which serves not only to entertain but to impart information, stimulate affective connection, encourage critical thought processes and introduce new perspectives (Rossiter *et al.* 2008). It can act as an avenue for engaging communities in embodied learning, reconfiguring understanding and stimulating empathy (Bunn *et al.* 2020). Theatre has been utilised widely in sectors such as human health to communicate messages on emerging topics of public health importance including HIV transmission and antibiotic resistance (Harvey *et al.* 2000; Mitchell *et al.* 2001; Ahmed *et al.* 2020) and to explore and challenge perceptions surrounding complex or sensitive subjects, including stigmas attached to mental health (Michalak *et al.* 2014; Nichols *et al.* 2022). Its accessible nature has led to its widespread use in LMICs especially to engage ‘hard to reach’ communities. However, in the field of animal welfare, theatre remains a rarely utilised approach.

Theatre’s wide general appeal also makes it suitable for engaging audiences of a broad age range, including young people and children. Theatre can appeal to the innate creativity of children and stimulate imaginations in a way that is unique (Eluyefa 2017). Involving children in theatre can be an effective way of facilitating their learning, and both cognitive and language benefits have been demonstrated (Eluyefa 2017; Furman 2000). In many contexts within which working equids are utilised, handling and supervision of the animal at work is routinely carried out by children (Swann 2006). For this reason, and the fact that today’s children will grow up to be the next generation of working equid owners, we chose to use theatre that could be adapted flexibly for children, in order to explore the use of theatre to convey targeted positive welfare messaging for a youth audience.

This study aimed to evaluate the potential of forum theatre as a tool for inclusive community engagement of both adults and children with positive donkey welfare messaging. Forum theatre is a type of theatre that highlights community issues, encourages interaction with the audience and discourse on how issues could be solved. The study is (to the authors’ knowledge) novel in its participatory development of a forum theatre production on donkey welfare and in its subsequent evaluation.

## Materials and methods

### Study location

Lamu Island is part of the Lamu archipelago, situated off the Kenyan coast. Lamu Old Town is the oldest and best-preserved Swahili settlement in East Africa and, as such, is a designated UNESCO world heritage site (UNESCO 2001). The narrow streets prevent the use of motorised vehicles and cars are banned on the island; as such, the main form of land transport, both of goods and people, is by donkey. Local communities are heavily reliant upon working donkeys for their livelihoods and many industries, including construction, agriculture and tourism, are dependent on the use of working donkeys. The donkey is also a symbol that has become synonymous with Lamu itself and a connection to the archipelago’s historic Swahili culture and practices. International NGO, The Donkey Sanctuary, has an established presence on the island, having worked there since 1987 and running a clinic which provides free access to equine veterinary services. Clinical data have highlighted prevalent donkey welfare issues in Lamu including wounds, hoof problems and colic (Olum & Rachuonyo 2021), internal investigations have also emphasised that many donkey owners have received no formal advice or training on donkey health or welfare. The unique social, economic and cultural importance of donkeys to the communities of Lamu Island and the Lamu archipelago, and the demographics of the communities themselves make it an ideal place to explore the potential for community engagement and the relaying of welfare messages through the medium of theatre.

### Ethics

The University of Portsmouth’s Ethics Committee for the Faculty of Humanities and Social Sciences reviewed and approved the study (reference FHSS 2023-057). Informed consent (verbal or written dependent upon literacy) was gained from all adult participants involved in focus groups or performance evaluation, from the legal guardians of children involved in performance evaluation and informed assent gained from the children themselves prior to their participation.

### Co-creation

Many factors can influence the success of welfare initiatives within communities. Previous research has highlighted the importance of community participation, a focus on addressing local priority issues and utilising people from within a target community as key influences on initiative success (Haddy *et al.* 2023a). Consequently, this initiative was designed to be co-created with the community, highlighting their concerns and lived experiences. Focus groups were conducted with donkey owners over three days; 31 owners attended the focus group sessions and sessions lasted, on average, 85 min. Participants were recruited via opportunity sampling from an area where donkey owners gather, and they were recompensed for their time. Researchers wanted to ensure that participants (in this case donkey users) felt a sense of ownership over the creation process as well as the output, thus maximising the likelihood that the message within would change mindsets and behaviours. As such, the focus groups aimed to ascertain from an owner perspective: how donkeys are perceived on Lamu; what they believed were the most prevalent and serious donkey welfare issues; the main barriers existing to improving donkey welfare; and any potential solutions to these that they could think of. Welfare issues identified by donkey owners as being present in the community included overloading of packs, some individuals beating donkeys, many donkeys with poor body condition and the ingestion of plastic causing (often fatal) impaction colic. These issues of welfare concern did largely coincide with welfare issues identified by The Donkey Sanctuary’s clinical data. However, discussion also included socioeconomic influences on donkey welfare and ownership which included rapidly increasing animal feed prices, increasing competition for employment, the long-term financial implications of selling a donkey and the potential of forming a donkey owner association in order to have more of a voice in local decision-making forums. Participants were then asked which topics that had been discussed during the session they most wanted to see represented in the drama production. Focus groups were facilitated by OS in Swahili, the participants’ first language and translated throughout into English in order for notes to be taken by EH.

### Dramatisation

Forum theatre is a particularly flexible and interactive type of theatre developed by Augusto Boal as part of a suite of techniques known as Theatre of the Oppressed (Boal 1979) which utilise theatre as an instrument for social change. Forum theatre has gained popularity as a pedagogical tool due to its ability to inspire reflection, creativity and vulnerability (Van Bewer *et al.* 2021). Forum theatre encourages audience participation by staging a narrative around characters who experience struggles within their community, based upon real life experiences, ultimately leading to a ‘bad ending’ for the characters. Once acted through, the play is then performed again but with audience members invited to join the cast to intervene at key points in the narrative, suggesting alternative choices that could have been made to bring about a different, more positive, outcome. This enables engagement in a way that empowers people to formulate their own solutions to the challenges presented and voice what will bring benefits to the local community. Due to the increased level of audience involvement, and the potential for stimulation of discussion, thought and knowledge sharing around the focal issue, forum theatre was chosen as the staging method for this study.

The drama piece was co-developed and performed by Lamu-based theatre group, Lamu Arts and Theatre Alliance (LATA), who have experience using forum theatre as a technique to explore sustainability and social justice issues in Lamu and the surrounding islands. The finalised play involved a cast of nine actors and an MC or ‘Joker’ and followed the story of a family whose livelihood relied upon their donkeys and the decisions that they made when facing challenging circumstances. In forum theatre, the relationship between the actors and the audience members is facilitated by a ‘Joker’, and this is a pivotal role.

The play utilised the main themes and topics raised in the focus groups, and welfare issues identified as locally common and problematic through The Donkey Sanctuary’s clinical data. The aspects of donkey welfare and management that featured throughout the play included: not overloading or beating donkeys; provision of adequate food, water and rest; seeking prompt veterinary treatment if an animal shows signs of illness; protection against and impacts of ingesting plastic waste. Before the play was performed to the public a number of donkey owners were invited to preview the production in order to check that they were comfortable with the content included and felt that it accurately represented the issues that they raised. As the scenes were scripted to end badly for the characters, the consequences of poor donkey welfare, for both donkeys and people were explored. The play was performed to the public in three locations during November and December 2023: Gadeni Village; Mkomani Village; and one performance at the Annual Lamu Cultural Festival in Lamu Old Town (Explore Lamu 2024). Performances were conducted outdoors in communally accessible spaces; no limit was placed on the number of people able to attend and viewing was free of charge. The duration of the play was approximately 30 minutes.

In the convention of forum theatre, the play was acted through twice and in the second performance audience members were invited to intervene at any point where they believed an alternative choice could have been made which would have resulted in a different outcome for the characters. In this way the production aimed not only to raise awareness of aspects of donkey welfare, but also to open up a dialogue with the audience to consider and play out alternative behaviours in order to achieve a different outcome. Audience members were able to come onto the stage and take on the role of one of the characters, while the other actors remained in character and improvised their reactions and responses. Interventions from the audience (although not necessarily leading to a better outcome for the donkey) stimulated further discussion and interactions with the audience.

### Data collection (public performances)

To evaluate the effectiveness of the theatre piece in engaging the audience and disseminating welfare information, feedback from audience members was collected via evaluation questionnaires. The welfare challenges portrayed in the play were conceptualised in three overarching categories: health needs (covering prompt treatment seeking and the threat of plastic ingestion); welfare needs (covering beating, food and water provision); and how much donkeys should carry (covering overloading and the need for rest). As such, these categories were used as key indicators to assess the dissemination of welfare information. Due to the outdoor setting of performances where the audience was dynamic and turnover could be rapid, questionnaires were conducted only post-performance. Quantitative data were collected by members of LATA using Likert scales (1–5) and qualitative data collected via open questions, for full details see the Adult Questionnaire in the Supplementary material. Data were collected in Swahili, the first language of both audience members and data collectors. Questions were read aloud to participants and their answers recorded. Responses were then translated into English by an individual involved with the development of the performance to ensure full understanding of the context of the data.

### Content adaptation for performance in schools

The drama piece required adaptation for performance in schools. Some content that featured in the original play had a focus on sociopolitical issues such as local government decision-making, which was felt to be irrelevant for children. Instead, the content of the play was re-imagined into a new format. The context-specific welfare challenges raised in the original piece (overloading, beating, provision of adequate nutrition, water and rest, the threat of plastic ingestion and the need to seek veterinary attention when signs of illness first appear) were retained and formed the basis for the scenarios presented. However, this was enacted in a format more engaging for children involving a donkey puppet character and had a focus on donkey sentience, i.e. how the donkey felt in the different scenarios presented. The forum theatre format was still utilised, with children able to intervene after a scenario had played out in order to change the ending of the scenario. In order to maximise children’s attention and engagement, the play was divided into four scenarios with the re-play happening after each scenario rather than at the end of the entire play.

### Data collection (school performances)

To evaluate the effectiveness of the theatre piece in improving donkey-related knowledge and changing attitudes, feedback was collected via pre- and post-performance questionnaires. The three key indicator categories used for adult performances were retained but additional questions were asked to evaluate aspects of donkey sentience, which featured as more of a focus in the adapted school performances. Quantitative data were collected by members of LATA using Likert scales (1–5) and qualitative data via open questions, for full details see the Schools Questionnaire in the Supplementary material. Again, data were collected in Swahili and questions read aloud if necessary. Answers were later translated into English for data analysis.

### Data analysis

Data from both public and school performances were analysed in SPSS® version 28 (IBM Corporation® 2021). Descriptive statistics were calculated for Likert scale questions. Distribution of the data were assessed using Shapiro-Wilk tests and data were determined to be non-parametric. For school performance data, related samples Wilcoxon signed rank tests, Mann Whitney *U* tests and Spearman’s rank correlations were used to test for differences in pre- and post-performance scores, differences in pre-performance scores based on gender and correlations between pre-question scores, respectively.

Content analysis was used to analyse responses to open questions. Answers that contained common subject content were grouped together in order to identify areas of learning and themes of importance resulting from audience perceptions of the performances. Initial groupings and code generation were carried out manually by EH using different coloured cells in Microsoft Excel®. These were then reviewed by OS and revised between the two authors to determine a consensus.

## Results

### Public performances

The three public performances reached a combined audience of approximately 600 people. Approximately 100 people attended each of the Gadeni and Mkomani performances and more than 400 attended the performance at Lamu Cultural Festival. Due to the dynamic nature of the performances at outdoor venues, attendees dispersed quickly following the conclusion of the performance and retaining people to answer questionnaires was challenging. Consequently, post-performance data were collected from 42 adult audience members (an additional five incomplete responses were excluded) across the performances. Quantitative feedback from the three public performances, measured on five-point Likert scales, is presented in Table 1.

**Table 1.** Public performance quantitative feedback (Categories ‘Neither agree nor disagree’ and ‘Strongly disagree’ were not utilised by survey participants so do not feature in the table but were available as options on the scale)

Question	Strongly agree n (%)	Somewhat agree n (%)	Somewhat disagree n (%)
Did you enjoy the performance?	39 (93%)	3 (7%)	-
The play raised my awareness of the different welfare needs that donkeys have	38 (91%)	4 (9%)	-
The play raised my awareness of how much donkeys should carry	38 (91%)	4 (9%)	-
The play raised my awareness of how to keep donkeys healthy	37 (88%)	5 (12%)	-
The play raised my awareness of the role of donkeys in the community	37 (88%)	5 (12%)	-
I think that using research-based drama is an effective way of changing people’s knowledge about donkeys	39 (93%)	3 (7%)	-
I think that using research-based drama is an effective way of changing people’s behaviour towards donkeys	39 (93%)	2 (5%)	1 (2%)

Qualitative results provided more in-depth information about audience perspectives. In response to the question “*how did the production make you feel?*”, 40% of participants (n = 17) indicated positive emotions (using the words happy/happiness or feeling good/nice). Others indicated that they felt empathetic emotions, these referenced feeling sorry for donkeys and the way they are treated (19%; n = 8). References were also made both to having learned something (17%; n = 7) and the play representing reality or truth (12%; n = 5).

In response to “*what was most helpful and/or interesting about the play and why?*”, information about how to take care of (or protect) donkeys was mentioned by 40% (n = 17) of participants. Other themes appeared to reflect the socioeconomic content of the play and included: the importance of donkeys (14%; n = 6), the idea of forming a donkey owners association (10%; n = 4), economic benefits associated with owning donkeys (7%; n = 3), the consequences of selling a donkey (7%; n = 3) and the education of children through the income made by working with donkeys (7%; n = 3).

In order to ascertain if there were any elements of the production that the audience did not like, participants were asked “*what was least helpful and/or interesting about the play and why?*” Some felt that all of the content was necessary or important and did not suggest any elements that were unhelpful (31%; n = 13). Behaviour of the characters portrayed or tensions between individuals (such as gossiping about other people, selfish behaviour and placing blame on others) was frequently mentioned (29%; n = 12). Other themes included: content featuring aspects of neglect such as wounds, mistreatment, not providing veterinary care or undervaluing the donkeys (19%; n = 8); the play being too short (5%; n = 2); and the setting being too noisy (2%; n = 1).

Participants were asked “*what did you learn from the performance?*” Many responses included learning about how to (or the need to) take care of donkeys and not mistreat them (48%; n = 20). Other areas of learning identified were the importance of donkeys to the community, including the reliance people have on them daily as an income source (24%; n = 10) and the benefits of donkeys (5%; n = 2).

In response to “*would you like to see more theatre productions for community messaging or would you prefer other methods such as talks, leaflets, radio?*” the majority of participants indicated a preference for drama (74%; n = 31). Others preferred: multiple media (12%; n = 5); lectures (5%; n = 2); television (5%; n = 2); flyers (2%; n = 1); radio (2%; n = 1). It should be noted that some participants suggested that the content of the play be adapted to other forms of media, such as television and radio, in order to reach more people.

Finally, participants were asked “*Do you have any feedback or ideas about how the production could be improved?*” Many participants said that no improvements were needed (38%; n = 16) but improvements suggested included: doing more performances or performances in other areas to reach more people (21%; n = 9); the addition of more information on taking care of donkeys (7%; n = 3); and that the play should be longer (7%; n = 3).

### School performances

The adapted play was performed by LATA in four secondary schools (two boys’ schools and two mixed schools) on the island during July and August 2024. A total of 120 young people participated, 82 boys (68%) and 38 girls (32%) with ages ranging from 11–23 (mean [± SD]: 16 [± 2] years). (In this context it is not uncommon for young people to return to complete their secondary schooling after the age of 18). Quantitative feedback from questions measured both before and after the performances is presented in Table 2.

**Table 2.** Quantitative feedback pre- and post-performance from four school performances (Five-point Likert scale measurement). Questions in bold indicate statistically significant differences in pre- and post-performance scores

Question	1. Strongly Agree n (%)	2. Somewhat Agree n (%)	3. Neither agree nor disagree n (%)	4. Somewhat Disagree n (%)	5. Strongly Disagree n (%)
*Do you like donkeys?Pre:	**110 (92%)**	**9 (7%)**	**1 (1%)**	**-**	**-**
**Post**:	**116 (97%)**	**4 (3%)**	**-**	**-**	**-**
Do you think donkeys are important?Pre:	114 (95%)	5 (4%)	1 (1%)	-	-
Post:	115 (96%)	5 (4%)	-	-	-
*Do you feel confident identifying how a donkey is feeling?Pre:	**83 (70%)**	**25 (21%)**	**2 (2%)**	**4 (3%)**	**5 (4%)**
**Post:**	**95 (79%)**	**22 (18%)**	**1 (1%)**	**-**	**2 (2%)**
Do you feel confident identifying when a donkey is not well?Pre:	89 (76%)	21 (18%)	3 (2%)	2 (2%)	2 (2%)
Post:	99 (82%)	19 (16%)	-	1 (1%)	1 (1%)
How much do you agree with the following sentences:					
You should load as much as possible onto your donkeyPre:	5 (4%)	7 (6%)	3 (3%)	5 (4%)	100 (83%)
Post:	8 (7%)	4 (3%)	2 (2%)	3 (2%)	102 (86%)
Donkeys need restPre:	112 (93%)	4 (3%)	2 (2%)	-	2 (2%)
Post:	115 (97%)	3 (2%)	1 (1%)	-	-
*Donkeys feel painPre:	**74 (62%)**	**5 (4%)**	**1 (1%)**	**-**	**40 (33%)**
**Post:**	**107 (90%)**	**3 (2%)**	**2 (2%)**	**1 (1%)**	**6 (5%)**
Donkeys feel emotionsPre:	102 (85%)	11 (9%)	2 (2%)	1 (1%)	4 (3%)
Post:	104 (87%)	11 (9%)	2 (2%)	2 (2%)	-
Donkeys need to be beaten to work hardPre:	11 (9%)	6 (5%)	6 (5%)	5 (4%)	90 (77%)
Post:	6 (5%)	6 (5%)	4 (3%)	9 (8%)	94 (79%)

Post-performance, young people were asked two additional quantitative questions. The majority of participants (98%; n = 117) strongly agreed that they enjoyed the performance with the remainder somewhat agreeing. The majority (92%; n = 107) also strongly agreed that theatre is an effective way of changing people’s behaviour towards donkeys.

Pre- and post-performance answers for nine questions (Table 2) were compared to determine whether the performance had made a statistically significant change in young people’s attitudes to or knowledge about donkeys. Statistically significant differences were seen for the questions concerning: whether participants liked donkeys (*P* = 0.05, both pre- and post-median = 1); whether they felt confident identifying how a donkey is feeling (*P* = 0.006, pre- and post-median = 1); and whether they thought donkeys feel pain (*P* < 0.001, pre- and post-median = 1). In these instances, more participants indicated that they liked donkeys, felt more confident identifying how donkeys were feeling and believed that donkeys felt pain after having watched the performance. No significant differences were found for the questions concerning: whether participants thought donkeys were important (*P* = 0.59, pre- and post-median = 1); whether they felt confident identifying when a donkey was unwell (*P* = 0.09, pre- and post-median = 1); agreement that as much as possible should be loaded onto a donkey (*P* = 1, pre- and post-median = 5); belief that donkeys need rest (*P* = 0.08, pre- and post-median = 1); belief that donkeys feel emotions (*P* = 0.16, pre- and post-median = 1); and belief that donkeys need to be beaten to work hard (*P* = 0.12, pre- and post-median = 5).

Some differences in pre-performance scores were seen based on participant gender. Girls indicated that they felt less confident identifying what a donkey was feeling (*P* = 0.006, median both M&F = 1) and less confident identifying when a donkey was unwell (*P* = 0.009, median M&F = 1) in comparison to boys. There was also a significant difference in belief that donkeys feel emotions (*P* = 0.04, median M&F = 1) with more boys believing that donkeys feel emotions than girls. However no significant differences were seen between genders in liking donkeys (*P* = 0.57, median M&F = 1), thinking they were important (*P* = 0.06, median M&F = 1), agreement with overloading (*P* = 0.41, median M&F = 5), need for rest (*P* = 0.67, median M&F = 1), belief in pain (*P* = 0.80, median M&F = 1) and agreement with beating (*P* = 0.57, median M&F = 5).

There was no significant correlation between participant age and enjoyment of the play (r = 0.12; *P* = 0.21). However, significant correlations were found between pre-performance belief that donkeys feel emotions and confidence in identifying what a donkey is feeling (r = 0.43; *P* < 0.001) and in identifying when a donkey is unwell (r = 0.44; *P* < 0.001). A significant correlation was seen for belief that donkeys feel pain and confidence in identifying what a donkey is feeling (r = –0.24; *P* = 0.009) although no significant correlation was found between belief in pain and confidence in identifying when a donkey was unwell (r = –0.15; *P* = 0.12).

### Qualitative feedback

In response to the question “*how did the production make you feel?*” two key emotional responses were identified. Many participants (48%; n = 57) cited positive emotional responses to the play, including happiness and enjoyment but also entertainment, excitement and motivation. Participants also reported feelings of empathy towards donkeys (23%; n = 27) most commonly sadness, pity and feeling bad on behalf of donkeys due to their poor treatment. The other identified themes centred around references to donkeys being important (4%; n = 5) and having a right to care and good treatment (11%; n = 13).

In response to being asked “*what did you learn from the performance?*” the most common answer mentioned the word ‘care’ in the context of needing to take care of donkeys (33%; n = 40). Other themes centred around the topics covered in the play, such as not beating or mistreating donkeys (13%; n = 15) or learning that donkeys feel pain or emotions (9%; n = 11). Some participants cited learning that donkeys are important (10%; n = 12), have rights (9%; n = 11) or need protection (3%; n = 4).

The final question asked “*Do you have any feedback or ideas about how the production could be improved?*” Two themes related to performing the production more widely; one to take the production out to the community and society more widely (21%; n = 25) and one to perform it in more schools (9%; n = 11). Other suggestions related to adding more educational content about donkeys (10%; n = 12) and including the young people in the performance more (3%; n = 3). Many participants also commented on theatre being a good way to educate people or an easy way to pass on an intended message (14%; n = 17).

## Discussion

Reactions to the theatre productions were positive with 93% of adult and 98% of youth audiences strongly agreeing that they enjoyed the performance. For adults, more than 85% of respondents strongly agreed that the performance raised their awareness of each of three key indicators: donkey health needs; donkey welfare needs; and how much donkeys should carry. For youth audiences, comparison of pre- and post-performance measures demonstrated positive changes in the belief that donkeys feel pain, how much individuals liked donkeys and how confident they felt in identifying how a donkey was feeling.

In feedback, both adult and youth audiences expressed the desire to see the production performed to more people in the community and the majority of adult participants (74%) indicated a preference for future community messaging to be delivered via theatre over other communication methods. The high acceptability of theatre to audiences has also been demonstrated in Uganda with a preference for live drama over videos as these were considered more ‘real’ and engaging (Mitchell *et al.* 2001). Using comparable data, evaluations of these live performances showed that compared to leaflets, community educators and videos, the plays reached larger numbers of people and a wider demographic (in terms of different sections of the community) more successfully than the other methods. However, it was highlighted that sometimes the intended ‘take home’ message was not that interpreted by audience members (Mitchell *et al.* 2001). Evaluative feedback in our study, particularly that related to the main areas of learning, highlighted: ‘the importance of donkeys’ and ‘donkey care’ for adults and the ‘need to care for donkeys’, ‘not to beat donkeys’ and that ‘donkeys feel pain and emotions’ for young people, all of which suggest that the take home messages were correctly received by audience members. However, in some instances, feedback indicated that the negative interactions between characters in the play may have distracted from the main welfare messaging.

For youth audiences, ceiling effects were seen across many questions which does indicate a generally good baseline of welfare knowledge in this population, however these ceiling effects may have limited the ability to show significant attitude changes in response to the play across these measures. Belief in donkeys’ ability to feel pain was the most polarised measure with only 62% of young people initially strongly agreeing that donkeys can feel pain. This may indicate a genuine lack of knowledge within the community that donkeys feel pain; donkeys are renowned for their stoicism and often display subtle pain cues which may prove difficult to read for inexperienced handlers or bystanders (Burden & Thiemann 2015). Interestingly, belief in donkey ability to feel emotions appeared to be treated separately with a higher baseline belief demonstrated (85%). Owner belief in both donkey pain and emotions has been linked to better donkey welfare (Haddy *et al.* 2023b) and, with this in mind, the youth theatre production centred on how the main donkey character was feeling in different scenarios and included a focus on not mistreating donkeys (as it causes them pain). It appears that these topics were effective in changing certain aspects of knowledge and attitudes with positive changes demonstrated between pre- and post-performance across measures of how much individuals liked donkeys, how confident they felt in identifying how a donkey was feeling and the belief that donkeys feel pain.

Differences were seen between genders in the level of confidence felt identifying how a donkey was feeling and when the animal was unwell, with girls scoring themselves as less confident than boys. This may be due to girls having less exposure to donkeys than boys; on Lamu it is still typically men and boys that work with donkeys and, as such, have closer interaction with them. Confidence levels though were correlated with belief in donkey emotions, with those more confident in identifying how a donkey was feeling and when the animal was unwell more likely to believe that donkeys feel emotions. Given the previously established relationship between sentience awareness and improved welfare, this could represent an area of concern regarding welfare. However, it also represents an opportunity for information dissemination to create an attitude change and, as such, targeting information for girls (or at a minimum ensuring the inclusion of girls) in equid welfare initiatives is strongly recommended. This mirrors the call for more women to be afforded access to extension services and training regarding working equids as women are often their primary caretakers although not their primary work handlers (Valette 2014).

Animal welfare-specific activities and initiatives for children and young people have been developed with a range of aims including decreasing incidences of animal cruelty, to promote safe and respectful interactions and creating awareness of species-specific welfare problems (Lakestani & Donaldson 2015; Hawkins *et al.* 2019; Williams *et al.* 2022). As in this theatre production, many of these activities place particular emphasis on the development of empathy towards animals and increasing children’s awareness that animals are sentient beings. There is increasing evidence that correlations exist between awareness of animal sentience and the treatment of animals across species (including working equids) with owners that exhibit greater awareness of animal sentience owning animals in better welfare states or employing more welfare-friendly working practices (Kielland *et al.* 2010; Luna *et al.* 2018; Bukhari *et al.* 2023; Haddy *et al.* 2023b). Therefore, the development of sentience-based educational content for children may provide an avenue to grow a more responsible next generation of people owning and interacting with animals. For working equids, who spend a large proportion of their time interacting with their handler, the quality of human-animal interaction plays a large part in both their physical and mental well-being (Luna & Tadich 2019) and education for school-aged children has been recommended as a strategy to increase future working equid welfare through improved human-animal relationships (Luna & Tadich 2019; Grace *et al.* 2022). NGOs, such as The Donkey Sanctuary and Brooke, run after-school ‘donkey care clubs’, which use a variety of activities to engage children in discussions about animal welfare including creative, arts-based learning and drama. The staging of drama has been described in children’s projects in Honduras and in Ethiopian schools to promote empathy for animals (Bojia *et al.* 2010; Bland 2014). Performances in Ethiopia involved portrayal of typical community practices and a comparison between a wise owner and an owner with poor practices and attitudes towards donkeys (Bojia *et al.* 2010). In this study, young people’s belief that donkeys feel pain was significantly improved after having watched the performance, indicating that theatre may be an effective tool for sentience education. Additionally, there was no difference in enjoyment of the play by age (11–23 years) which appears to demonstrate suitability of theatre to engage young people across the secondary school age range and above.

Creation of the play’s content in conjunction with donkey owners (through focus group discussions and pre-performance feedback) meant that owners were able to see issues of importance to them represented to the wider community. Therefore, receiving audience feedback that the play represented ‘reality’ or ‘truth’ was pleasing as creation of a sense of ownership over an initiative is key to its sustainability and success (Wabwoba & Wakhungu 2013; Ceptureanu *et al.* 2018). It is acknowledged that participatory approaches (including arts-based methods) come with their own inherent issues of power dynamics both within communities and between participants and researchers (Cooke & Kothari 2001; Chinyowa 2015). Although researchers were inevitably positioned as ‘outsiders’ throughout the research process, it was ensured that any ‘solutions’ to issues depicted in the play came from the community solving its own problems and not from outside intervention. The use of local artists to produce the play also helped to ensure that the content was specific to the local context and aided in creating a dynamic of trust with the community.

Parallels were seen across adult and youth feedback, clustering of both positive and empathetic emotions were seen when describing how the production made people feel. A key strength of theatre is its ability to induce emotional engagement with its content, communicating with audiences on an affective and cognitive level (Kontos & Naglie 2007; Michalak *et al.* 2014). Emotional responses to material portrayed in research-based theatre (such as being moved to tears) has been reported alongside generation of new awareness, knowledge and understanding of the target group (Nichols *et al.* 2022). In this study, those that felt empathetic emotions described feeling sad for donkeys, indicating emotional identification with the donkeys themselves, which may open up new critical reflection and ways of thinking.

Although there is evidence that the production has changed young people’s knowledge about and attitudes towards donkeys, we must be cautious about the assumption that this will translate into behaviour change. Sometimes referred to as the attitude-behaviour gap, discrepancies between pro-welfare attitudes and pro-welfare actions have been demonstrated (Cornish *et al.* 2019). Models of behaviour change, such as the COM-B model, suggest that there are many factors that influence human behaviour and thus a person’s ability to carry out a pro-welfare action, including capability, opportunity and motivational components (Michie *et al.* 2011). Behaviour changes resulting from health-related theatre initiatives have been demonstrated (Harvey *et al.* 2000; Middelkoop *et al.* 2006), however evidenced examples are not common. Whilst an arts-initiative alone is unlikely to be able to tackle the root causes of problematic behaviour within a community, there is scope for arts-based methods to work well in conjunction with other initiative types and multi-approach initiatives have been highlighted as successful in creating sustainable welfare outcomes (Haddy *et al.* 2023a). Due to its ability to reach a large number of people, theatre is an ideal method of starting a conversation (particularly with communities that may not be especially trusting or receptive) which can then be built upon by other context-appropriate approaches.

Despite theatre frequently being used as a medium for public health information dissemination in LMICs, especially in sub-Saharan Africa, much of the formal theatre evaluation literature is based on examples from high income countries (Bunn *et al.* 2020). To the authors’ knowledge this study is the first to evaluate the success of an animal welfare theatre initiative within an LMIC context. In future, a larger-scale initiative which included pre- and post-performance data for adults would give improved rigour in the evaluation process. Staging the performances outdoors meant that they were accessible to large numbers, but this was also a limitation to evaluation, as the dynamic, open audience did not allow for controlled selection of participants. Medium- and long-term follow-up questionnaires would be useful to determine sustained impact of the performances.

### Animal welfare implications

The question of how to change human behaviour to create a better life for animals is the key question underlying the field of animal welfare. Although there are inherently many challenges to changing human behaviour, one of the most basic is connecting with people and engaging them with animal welfare information. For ‘hard to reach’ communities this can be even more difficult, and we need to find methods of communication that are culturally suitable and accessible to all community members. This study evaluates the feasibility of using participatory theatre as a methodology for engaging donkey-reliant communities with welfare information. Arts-based methods are not widely used in the animal welfare field and, as such, this study provides valuable information on the potential of these methods to be an avenue for disseminating positive welfare information and stimulating discussion with communities. The findings are applicable beyond just donkey welfare and have direct relevance to animal welfare practitioners, especially those working with ‘hard to reach’ communities.

## Conclusion

The study highlights the potential value of participatory theatre in community engagement for positive change in animal welfare across both adults and young people. The production was positively received, with 93% of adult and 98% of youth audiences strongly agreeing that they had enjoyed the performance. For adults, more than 85% of respondents strongly agreed that the performance raised their awareness of three key learning indicators: donkey health needs; donkey welfare needs and how much donkeys should carry. For youth audiences, comparison of pre- and post-performance measures demonstrated significant positive changes in the belief that donkeys feel pain, how much individuals liked donkeys and how confident they felt in identifying how a donkey was feeling. Qualitative feedback indicated that the production elicited empathetic emotions such as feeling sad for donkeys, with empathy considered to be a key attitudinal factor associated with better animal welfare. Audiences also expressed a desire for the production to be performed more widely within the community. Despite remaining rare in the animal welfare sector, arts-based initiatives have wide general appeal, can reach large numbers of individuals and break down barriers. As such arts-based methods could be especially useful in communities where challenges to more ‘traditional’ forms of information dissemination exist.

## Supporting information

Haddy et al. supplementary material Haddy et al. supplementary material

## Supplementary material

The supplementary material for this article can be found at http://doi.org/10.1017/awf.2025.12.
