# Supplementary material for: Forum theatre as a tool to promote positive donkey welfare on Lamu Island, Kenya
Source: Anim Welf. 2025 Mar 14;34:e16. doi: 10.1017/awf.2025.12 (PMC11936729; doi:10.1017/awf.2025.12)
Supplement: Haddy et al. supplementary material [file S0962728625000120sup001.pdf]

# Forum theatre as a tool to promote positive donkey welfare on Lamu Island, Kenya

E Haddyhttps://orcid.org/0000-0001-6696-0088[1], L Proops[2], T Bradley[1], C Bowyer[3], O Sing'Oei[4]

[1] Faculty of Humanities and Social Sciences, University of Portsmouth, Portsmouth, UK

[2] Centre for Comparative and Evolutionary Psychology, School of Psychology, Sport and Health Sciences, University of Portsmouth, Portsmouth, UK

[3] Faculty of Creative and Cultural Industries, University of Portsmouth, Portsmouth, UK

[4] The Donkey Sanctuary, Lamu Island, Kenya

Author for correspondence: Emily Haddy, email: emily.haddy@port.ac.uk

## Supplementary material

### Performance Evaluation Questionnaire

**1) Did you enjoy the performance?**

1. Strongly Agree
2. Somewhat Agree
3. Neither Agree nor Disagree
4. Somewhat Disagree
5. Strongly Disagree

**2) How did the production make you feel?**

**3) What was most helpful and/or interesting about the play, and why?**

**4) What was least helpful and/or interesting about the play, and why?**

**5) What did you learn from the performance?**

**6) Please indicate the extent to which you agree with the following statements.**

- **The play raised my awareness of the different welfare needs that donkeys have**
  1. Strongly Agree
  2. Somewhat Agree
  3. Neither Agree nor Disagree
  4. Somewhat Disagree
  5. Strongly Disagree

- **The play raised my awareness of how much donkeys should carry**
  1. Strongly Agree
  2. Somewhat Agree
  3. Neither Agree nor Disagree
  4. Somewhat Disagree
  5. Strongly Disagree

- **The play raised my awareness of how to keep donkeys healthy**
  1. Strongly Agree
  2. Somewhat Agree
  3. Neither Agree nor Disagree
  4. Somewhat Disagree
  5. Strongly Disagree

- **The play raised my awareness of the role of donkeys in the community**
  1. Strongly Agree
  2. Somewhat Agree
  3. Neither Agree nor Disagree
  4. Somewhat Disagree
  5. Strongly Disagree

**7) Would you like to see more theatre productions for community messaging or would you prefer other methods such as talks, leaflets, radio?**

**8) I think that using research-based drama is an effective way of changing people's knowledge about donkeys?**

1. Strongly Agree
2. Somewhat Agree
3. Neither Agree nor Disagree
4. Somewhat Disagree
5. Strongly Disagree

**9) I think that using research-based drama is an effective way of changing people's behaviour towards donkeys?**

1. Strongly Agree
2. Somewhat Agree
3. Neither Agree nor Disagree
4. Somewhat Disagree
5. Strongly Disagree

**10) Do you have any feedback or ideas about how the production could be improved?**

**Child Assent Script:**

The people you have met are working on a study to see how theatre can affect the way people think about donkeys. The next part of the study is for you to answer some quick questions about your thoughts on donkeys. You will then watch a play about donkeys and how people treat them. After the play, you will answer some questions about what you thought of the play.

Do you feel okay about carrying on with the study?

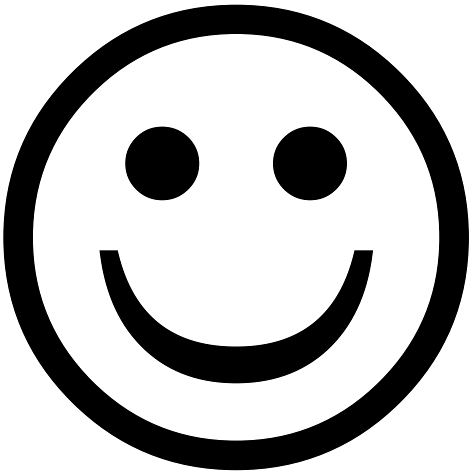

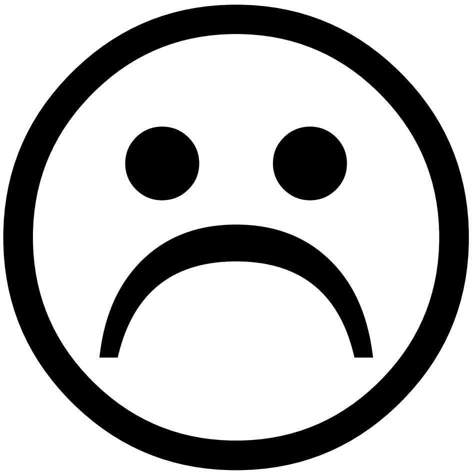

Yes

No

## Performance Evaluation Questionnaire (schools)

**Pre-performance questions**

- Do you like donkeys?
  1) Strongly agree
  2) Somewhat agree
  3) Neither agree nor disagree
  4) Somewhat disagree
  5) Strongly disagree

- Do you think donkeys are important?
  1) Strongly agree
  2) Somewhat agree
  3) Neither agree nor disagree
  4) Somewhat disagree
  5) Strongly disagree

- Why do you think donkeys are important or not important?

- Do you feel confident in identifying how a donkey is feeling?
  1) Strongly agree
  2) Somewhat agree
  3) Neither agree nor disagree
  4) Somewhat disagree
  5) Strongly disagree

- Do you feel confident in identifying when a donkey is ill?
  1) Strongly agree
  2) Somewhat agree
  3) Neither agree nor disagree
  4) Somewhat disagree
  5) Strongly disagree

- Can you list 3 things that donkeys need to keep them healthy?

How much do you agree with the following sentences?

- You should load as much as possible onto your donkey
  1) Strongly agree
  2) Somewhat agree

3) Neither agree nor disagree
4) Somewhat disagree
5) Strongly disagree

- Donkeys need rest
  1) Strongly agree
  2) Somewhat agree
  3) Neither agree nor disagree
  4) Somewhat disagree
  5) Strongly disagree

- Donkeys feel pain
  1) Strongly agree
  2) Somewhat agree
  3) Neither agree nor disagree
  4) Somewhat disagree
  5) Strongly disagree

- Donkeys feel emotions
  1) Strongly agree
  2) Somewhat agree
  3) Neither agree nor disagree
  4) Somewhat disagree
  5) Strongly disagree

- Donkeys need to be beaten to work hard
  1) Strongly agree
  2) Somewhat agree
  3) Neither agree nor disagree
  4) Somewhat disagree
  5) Strongly disagree

**Post-performance questions**

- Did you enjoy the performance?
  1) Strongly agree
  2) Somewhat agree
  3) Neither agree nor disagree

4) Somewhat disagree
5) Strongly disagree

- How did the production make you feel?

- What was your favourite part and why?

- What was your least favourite part and why?

- What did you learn from the performance?

- Do you like donkeys?
  1) Strongly agree
  2) Somewhat agree
  3) Neither agree nor disagree
  4) Somewhat disagree
  5) Strongly disagree

- Do you think donkeys are important?
  1) Strongly agree
  2) Somewhat agree
  3) Neither agree nor disagree
  4) Somewhat disagree
  5) Strongly disagree

- Why do you think donkeys are important or not important?

- Do you feel confident in identifying how a donkey is feeling?
  1) Strongly agree
  2) Somewhat agree

3) Neither agree nor disagree
4) Somewhat disagree
5) Strongly disagree

- Do you feel confident in identifying when a donkey is not well?
  1) Strongly agree
  2) Somewhat agree
  3) Neither agree nor disagree
  4) Somewhat disagree
  5) Strongly disagree

- Can you list 3 things that donkeys need to keep them healthy?

How much do you agree with the following sentences?

- You should load as much as possible onto your donkey
  1) Strongly agree
  2) Somewhat agree
  3) Neither agree nor disagree
  4) Somewhat disagree
  5) Strongly disagree

- Donkeys need rest
  1) Strongly agree
  2) Somewhat agree
  3) Neither agree nor disagree
  4) Somewhat disagree
  5) Strongly disagree

- Donkeys feel pain
  1) Strongly agree
  2) Somewhat agree
  3) Neither agree nor disagree
  4) Somewhat disagree
  5) Strongly disagree

- Donkeys feel emotions
  1) Strongly agree

2) Somewhat agree
3) Neither agree nor disagree
4) Somewhat disagree
5) Strongly disagree

- Donkeys need to be beaten to work hard
  1) Strongly agree
  2) Somewhat agree
  3) Neither agree nor disagree
  4) Somewhat disagree
  5) Strongly disagree

- Do you think that theatre is an effective way of changing people's behaviour towards donkeys?
  1) Strongly agree
  2) Somewhat agree
  3) Neither agree nor disagree
  4) Somewhat disagree
  5) Strongly disagree

- Do you have any ideas about how the performance could be improved?
